# Effect of Myosin Isoforms on Cardiac Muscle Twitch of Mice, Rats and Humans

**DOI:** 10.3390/ijms23031135

**Published:** 2022-01-20

**Authors:** Momcilo Prodanovic, Michael A. Geeves, Corrado Poggesi, Michael Regnier, Srboljub M. Mijailovich

**Affiliations:** 1 Institute for Information Technologies, University of Kragujevac, 34000 Kragujevac, Serbia; momcilo.prodanovic@kg.ac.rs; 2 Bioengineering Research and Development Center (BioIRC), 34000 Kragujevac, Serbia; 3 FilamenTech, Inc., Newtown, MA 02458, USA; 4 Department of Biosciences, University of Kent, Canterbury CT2 7NJ, Kent, UK; m.a.geeves@kent.ac.uk; 5 Department of Experimental & Clinical Medicine, University of Florence, 20134 Florence, Italy; corrado.poggesi@unifi.it; 6 Department of Bioengineering, University of Washington, Seattle, WA 98105, USA; mregnier@uw.edu; 7 Department of Biology, Illinois Institute of Technology, Chicago, IL 60616, USA

**Keywords:** MUSICO platform, cross-species simulation, trabeculae, level of incorporation, tension relaxation, calcium sensitivity

## Abstract

To understand how pathology-induced changes in contractile protein isoforms modulate cardiac muscle function, it is necessary to quantify the temporal-mechanical properties of contractions that occur under various conditions. Pathological responses are much easier to study in animal model systems than in humans, but extrapolation between species presents numerous challenges. Employing computational approaches can help elucidate relationships that are difficult to test experimentally by translating the observations from rats and mice, as model organisms, to the human heart. Here, we use the spatially explicit MUSICO platform to model twitch contractions from rodent and human trabeculae collected in a single laboratory. This approach allowed us to identify the variations in kinetic characteristics of α- and β-myosin isoforms across species and to quantify their effect on cardiac muscle contractile responses. The simulations showed how the twitch transient varied with the ratio of the two myosin isoforms. Particularly, the rate of tension rise was proportional to the fraction of α-myosin present, while the β-isoform dominated the rate of relaxation unless α-myosin was >50%. Moreover, both the myosin isoform and the Ca^2+^ transient contributed to the twitch tension transient, allowing two levels of regulation of twitch contraction.

## 1. Introduction

The characteristics of the cardiac twitch are dependent on multiple, interrelated cardiomyocyte parameters such as the calcium (Ca^2+^) transient, the degree of sarcomere shortening during force development and the associated elasticity of the cardiac cell(s), as well as the details of the actin-myosin activation and contraction cycle. All these factors need to be accounted for to accurately simulate the twitch, which we recently demonstrated with a model of a twitch for the rat trabecula using an explicit 3D model based on the MUSICO platform [1]. This model was able to predict the tension transient for a single fixed-length sarcomere, in which no shortening occurred, and for the trabeculae held at a fixed overall length but within which significant internal shortening occurred at the sarcomere level. The latter simulations included multiple sarcomeres in series forming myofibrils and contained a passive elastic component, taking into account the alignment of myocytes and their myofibrils within the trabeculae. While this was a significant achievement, the outcomes of this model system do not simply translate to human myocytes because of differences in the isoforms of proteins present in the specific cardiac cell types. A primary difference is the presence of the myosin isoform, which greatly affects crossbridge cycling kinetics. The adult rat ventricle contains 75% α-myosin (MyHC6) [2,3], whereas the human ventricle is ~90–99% β-myosin [4,5]. The human atria are close to the rat ventricle at around 75% α-myosin [5,6,7].

The α- and β-myosin isoforms have quite distinct effects on the crossbridge cycle. Both rates of adenosine triphosphate (ATP) consumption (i.e., ATPase rates) and the velocity of F-actin over the myosin beds in a motility assay are two- to three-fold faster for the α-myosin isoform compared to β-myosin [8,9,10]. These differences are reflected in the contractile parameters measured in intact or skinned myocytes and in isolated myofibrils, though these myocytes differ by much more than just the myosin isoform present [11,12]. Furthermore, the same myosin isoforms from different mammals are also quite distinct: β-myosins from the mouse (1.71 ± 0.18 µm/s per half-sarcomere (hs)) and rat (1.42 ± 0.14 µm/s per hs) move actin faster than the human β-myosin (0.330 ± 0.022 µm/s per hs) [13]. In general, the velocity of contraction, rate of tension rise and rate of ATP turnover scale inversely with the size of the mammal. Fortunately, due to the relatively recent increase in the availability of human muscle myosin from in vitro expression systems, we know the details of how pure human α- and β-myosins differ, and how these same isoforms differ among the mouse, rat, rabbit and human.

In this current study, based on in vitro measurements, we extend our models of mouse and rat ventricular twitches to the human ventricle. An additional consideration is that the myosin isoform content of the human heart is not homogeneous. The ratio of α:β isoforms varies across the heart wall as well as between the ventricle and the atria [12]. The α:β ratio is also influenced by factors such as age, sex and health status [14]. The variability of the α:β ratio is much better defined in model experimental systems like the rat where the effects of exercise, pressure overload and thyroid state have been defined [12]. Therefore, it is of interest to understand the effects of variable α:β ratios of myosin on the characteristics of the cardiac twitch. Our explicit 3D model allows us to vary the α:β ratio present, and to consider how the isoforms are distributed, within sarcomeres. We recently demonstrated this approach for simulations of cardiac troponin C (cTnC) variants with different Ca^2+^-binding affinities in transgenic mice [15]. We simulated the effects of variable amounts of mutant cTnC and explored the effects of random vs. biased clustering of mutant vs. WT cTnC on mouse cardiac muscle twitches.

Here, we present a study of how twitch contractions for mouse, rat and human trabeculae expressing 100% α, 75% α and 100% β, respectively, can be simulated by adjusting key sarcomere parameters. We demonstrate that by using known myosin isoform characteristics and measured Ca^2+^ transients, it is possible to reproduce the tension transients of fixed-length trabeculae from rodents and humans. Furthermore, we explore the effect of varying the α:β ratio of myosin in each species by changing the content of the α-myosin isoform (from 0 to 100%) and assuming a fixed Ca^2+^ transient. Preliminary reports of this work have been published previously [16].

## 2. Simulation of Cardiac Muscle Twitch Contractions

Cardiac muscle contains atrial and ventricle cells that are about the same size, ~100–140 μm long and 15 × 20 μm in cross-section, have a similar overall sarcomere geometry and show almost no differences between mammalian species. Cardiomyocytes contract in the same range of sarcomere lengths, from 1.9 to 2.2 μm, and generate about the same peak force per myocyte of 10 to 15 μN, i.e., a maximum twitch peak tension in the range of 33–50 kPa [17]. A major difference is the proportion of α- and β-myosin isoforms, where, depending on the species, each isoform has distinct kinetic characteristics specific to the cardiac muscle contraction and relaxation rates associated with the animal’s or human’s heartbeat frequency.

**Figure 1:**
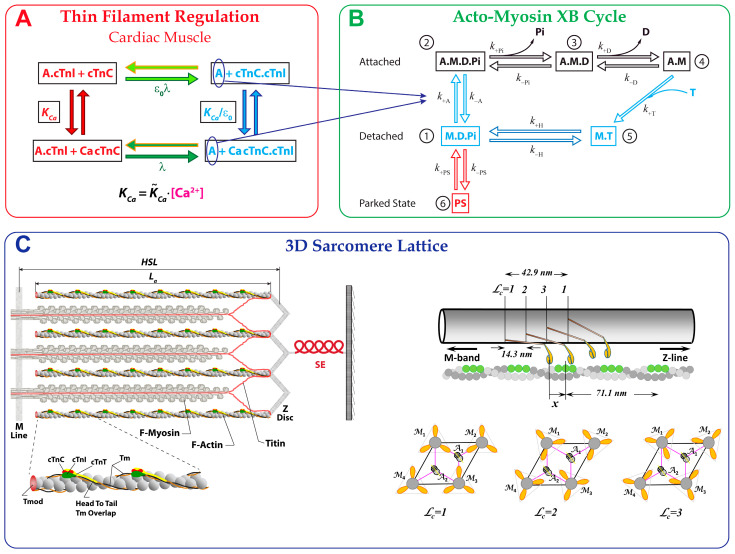
Schematic representation of MUSICO platform in reference to the 3D half-sarcomere structure. (**A**) Kinetic scheme of calcium-binding to cTnC and interaction of cTnI with actin in cardiac muscle. Calcium-binding to cTnC forms a CacTnC complex, with an equilibrium rate of $K_{Ca}={\tilde{K}}_{Ca}\cdot \left[Ca^{2+}\right]$, where ${\tilde{K}}_{Ca}$ is a rate constant and $\left[Ca^{2+}\right]$ is the instantaneous calcium concentration. Conformational changes within the cTn molecule forming the cTnC.cTnI or CacTnC.TnI complex, which have a low affinity to actin, are defined by the equilibrium state transition rate λ. In the CacTnC.cTnI (or cTnC.cTnI) state(s), cTnI is dissociated from actin, enabling Tpm to move freely, mostly azimuthally, and permitting myosin-binding and force generation. The rate of calcium-binding to cTnC.TnI to form a CacTnC.cTnI complex is accelerated by a factor of 1/ε_o_. (**B**) Six-state model of the actomyosin cycle includes five biochemical states consistent with observed structural states: M.D.Pi, A.M.D.Pi, A.M.D, A.M and M.T. The strain-dependent state transition rates are associated with conformational changes defining the structural conformations of myosin in each state (for details, see [1,15]). An additional sixth state, the so-called “parked state” (PS), represents the interaction of myosin heads with the thick filament backbone. The transition rate from PS to M.D.Pi is assumed to be strongly dependent on [Ca^2+^], and the reverse rate is independent of [Ca^2+^]. (**C**) The 3D structural organization of sarcomeres includes thick (myosin) filaments interdigitated with thin filaments composed of actin and regulatory proteins, along with the ancillary protein titin. The 3D formulation includes an azimuthal shift of myosin crowns (*L_c_* = 1, 2, 3), where the interaction of crossbridges with the target zones (*green filled circles*) on an actin filament is defined by axial crossbridge distortion and azimuthal angles defining the positions of actin filaments relative to the myosin filament and the positions of myosin-binding sites on actin (for details, see [21]). The parameters defining the lattice are the half-sarcomere length (*HSL*), the length of the actin filament (*L_a_*) and the interfilament spacing (*d_10_*). The trabecula elasticity (SE) in series with the contractile element (CE) consisting of multiple sarcomere muscle fibers [1] is symbolically displayed here as the SE and 3D half-sarcomere structure, respectively. The thin filament sketch is adapted from the fragment in figure of Gordon et al. [23] and the thick filament from Anderson and Granzier [24].

The MUSICO computational model was recently updated to simulate cardiac muscle twitch contractions with consideration of the explicit three-dimensional (3D) structure of a sarcomere (Figure 1). The model accounts for all interactions between sarcomere proteins, e.g., myosin with actin, the troponin-tropomyosin complex with the actin surface and proteins with small molecules, e.g., Ca^2+^ [18,19,20,21,22]. This approach is suitable for incorporating mixtures of myosin isoforms and protein mutations into computational models of twitch contractions of skeletal and cardiac muscles [1,10,15].

Specifically, in this study, we explore the effects of various amounts of α- and β-myosin isoforms on the contractile characteristics of cardiac muscle across muscle types and species.

The multiscale model consists of four major parts: (1) a detailed multi-sarcomere geometry for simulating myofibril contraction [21,22]; (2) a serial elastic element (SE) accounting for the changes in sarcomere length when the muscle is held at a fixed length [1]; (3) a six-state crossbridge cycle that includes a “parked state” [1,15], and (4) thin filament regulation by a four-state scheme for Ca^2+^-binding to cardiac troponin (cTn) coupled with a continuous flexible chain (tropomyosin) model for regulating myosin-binding to thin filament(s) [1,25].

### 2.1. Cardiac Muscle Structure

The explicit configuration of multi-sarcomeres in series contains 3D sarcomere structures where each half-sarcomere contains a regular array of thin and thick filaments in the myofilament lattice, which are connected by crossbridges and other elastic elements (e.g., titin [22]) in a structural network [1,15,21,26]. Each half of a thick filament consists of ~150 myosin molecules projecting from each side of the M-line. In cardiac muscle, the thin filaments are of variable lengths [27], with each having from 220 to 400 actin monomers emerging from Z-discs on opposite sides of a sarcomere. The thin filaments contain the regulatory proteins, tropomyosin (Tpm) and troponin (cTn), which are essential for Ca^2+^ regulation of contraction. The lattice also includes the auxiliary protein titin, which connects the Z-disc with the tip of the thick filament, and via the filament backbone, with the M-line (Figure 1C). For simulations of twitches in trabeculae at fixed lengths, the model includes the series elasticity (SE) derived from tension-displacement relations [28,29].

### 2.2. Six-State Crossbridge Cycle

The crossbridge cycle is as described in [1,15], and in brief, consists of five biochemical states of the chemomechanical cycle (Figure 1B), which are consistent with observed structural states: an M.D.Pi detached state (1), an A~M.D.Pi weakly bound state (2), an A.M.D strongly bound post-powerstroke state (3), an A.M rigor state (4) and an M.T detached state (5). In addition, the model contains a “parked” state (PS) (6) associated with thick-filament regulation (by Ca^2+^). The transition rates between the crossbridge states for the rat and mouse cardiac trabeculae were defined in Mijailovich et al. [1,15]. These rates are either independent or strongly dependent on the bond distortion (strain) except for the transition from PS into the M.D.Pi state, which was modeled as a [Ca^2+^]-dependent switch for myosin to a form that is available to bind actin. The mathematical formulation of the crossbridge cycle rates used here is adapted from [1,15].

### 2.3. Calcium-Regulated Tension Generation

Muscle contraction and relaxation are regulated by the Ca^2+^-dependent azimuthal movements of tropomyosin-troponin complexes over the surface of the actin filament. Structurally, tropomyosin (Tpm) covers seven monomers on one strand of the actin double helix and is associated with one cardiac troponin (cTn). cTn consists of troponin-T, troponin-C and troponin-I, denoted as cTnT, cTnC and cTnI, respectively. The Tpm-cTn azimuthal movements are defined by the position where Ca^2+^ binds with or dissociates from cTnC and changes the affinity of cTnI to actin by means of a conformational change in the cTn complex (inset in Figure 1C).

A minimal description of the allosteric mechanism in cardiac muscle includes four states: two cTnC closed states, where cTnC has no or one bound Ca^2+^ to N-terminal site II (denoted as cTnC and CacTnC, respectively), and, similarly, two cTnC open states (Figure 1A). In the presence of Ca^2+^, the open state is favored over the closed state and TnI binds preferentially to the open cTnC, showing a low affinity to actin. In the absence of Ca^2+^, the closed form of cTnC is favored and cTnI binds preferentially to actin. The kinetics of the interactions between the Tpm-cTn complexes with an actin filament are, at present, best described by a continuous flexible chain (CFC) model [25,30] that includes structural links between Tpm-cTn regulatory units (see inset in (Figure 1C) and [25]). These links are supported by structural evidence that neighboring Tpms overlap and that one end of cTnT is bound to a specific site on Tpm, whereas its N-terminus overlaps the adjacent Tpm and thus forms linked Tpm–Tpm regions. The interconnected neighboring Tpm-cTn units form a continuous flexible chain (CFC) on each strand of actin [31,32], rather than a set of independent Tpm-cTn units.

In the spatially explicit (3D) sarcomere lattice (Figure 1C) [21], active tension is obtained from all crossbridges bound to actin filaments. The evolution of bound crossbridge states is determined from coupled thin-filament regulatory processes and actomyosin cycle-state transitions. These transitions are stochastic processes defined by the standard Metropolis algorithm where a kinetic transition in a time step Δt occurs when a random number in (0, 1) lies in the range (0, kΔt), where k is the state transition rate constant. Setting Δt to be much smaller than the inverse of the fastest state transition rate in the kinetics process is sufficient to avoid interference between multiple transitions within a single subsystem (i.e., myosin filament interacting with surrounding actin filaments) and shows negligibly small interference between subsystems [1,21].

Overall, the Monte Carlo process is defined by two sets of random-number drawings within each time step: (1) the set of drawings that defines the transitions of cTnI-actin states from the transition rates between four states of the Ca^2+^ kinetics scheme; and (2) the set of drawings that defines the changes in actomyosin states modulated by the angular position of the CFC (for details, see [21,33]). The angular position of the CFC is defined by its mean angle and its azimuthal variation at the axial location of the binding site along the actin filament strand [25]. This CFC configuration is calculated after the first drawing based on the positions of current-bound cTnIs to actin and the positions of bound myosins from the previous time-step configuration. After completing both drawings, the spatial positions and states of all the bound crossbridges are established, and the crossbridge forces are calculated from the instantaneous mechanical equilibrium of all active forces, i.e., the sum of all bound crossbridge forces, with external forces and constraints.

### 2.4. MUSICO Predictions of Twitch Transients in Intact Trabeculae from Mice, Rats and Humans

In our previous work, we demonstrated that we could simulate a twitch tension transient of a trabecula at a fixed length for both the rat and mouse using the MUSICO platform (Mijailovich et al. [1,15]). Translation of the parameters between isoforms and species is not trivial; in fact, it is essential if data from model organisms such as transgenic mice are to be used to predict the behaviors of normal and pathogenic human cardiomyocytes. The MUSICO platform is a powerful tool that when using the explicit 3D structure of the sarcomere, can deal with mixed isoforms present in a single or multiple sarcomeres (see Mijailovich et al. [15]).

## 3. Results

**Figure 2:**
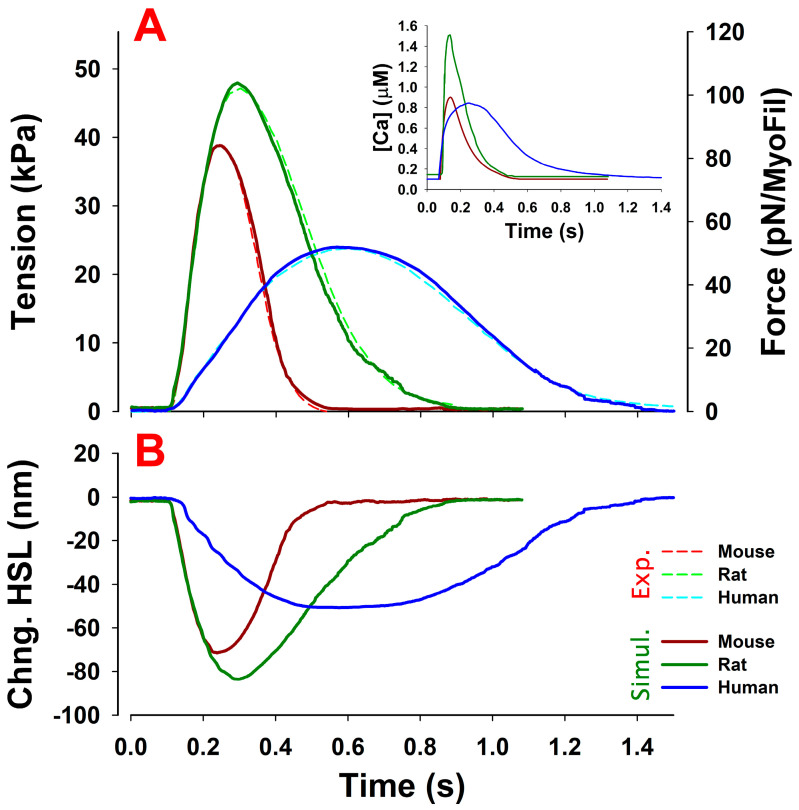
MUSICO predictions of twitch contractions in mouse, rat and human fixed-length trabeculae (**A**). The predicted tensions (thick solid lines) showed excellent agreement with those observed in mice, rat and human trabeculae (Chung et al. [34]) (*thin dashed lines*). The fits were achieved with a six-state crossbridge cycle that includes a parked state (PS) with calcium-dependent $k_{PS}$ and trabeculae series elasticity (SE). The crossbridge cycle state transition rates were for a completely α-cardiac myosin isoform in mice and a predominantly β-isoform in human simulations. In rats, the α- and β-isoform homodimers were randomly distributed, helically arranged myosin head pairs along a thick filament, containing an average of 75% α and 25% β. Following the experiments of Chung et al. [34], the simulations were done on fixed-length trabeculae at a relaxed sarcomere length of 2.2 μm and at 25 °C. The calcium transients (inset) were taken from rat right ventricle (RV) trabeculae at 25 °C (Janssen et al. [35]). The mouse calcium transient was derived from Ferrantini et al. [36] and the human calcium transients were taken from Ferrantini et al. [37]. The common model parameters are shown in Table 1 and parameters specific to mouse, rat and human trabeculae are shown in Table 2. MUSICO simulations also predicted a change in the half-sarcomere length, denoted as “Chng. HSL” in (**B**). The changes in the sarcomere length were not experimentally recorded in mice, rats or humans, but the predicted displacements from the rat simulations were similar to the observations of Caremani et al. [28] in rat fixed-length trabeculae.

Simulations of trabeculae twitches from the mouse, rat and human demonstrating good fits to the experimental twitch data are shown in Figure 2. In the following paragraphs, we describe how these simulations were obtained with a minimal set of changes in the crossbridge parameters between each species. The changes required between species are compatible with what is known about the properties of each myosin isoform. We then explored the effect of variation on the ratio of the two myosin isoforms (α and β) in a sarcomere on the twitch of each species, assuming the calcium transient remained the same in each case.

To achieve the simulations shown in Figure 2 several issues needed to be addressed. In the initial studies of rat and mouse twitches [1,15], we assumed a single population of myosin isoforms, which is correct for mouse trabeculae (100% α-myosin). However, the normal adult rat contains a 75%/25% mixture of α- and β-myosin while human trabeculae contain ~95% β-myosin. It was necessary, therefore, to understand the properties of the α- and β-myosin isoforms independently, the differences between each isoform in the three species and the behavior of a mixture of isoforms.

An additional challenge when modeling the twitches of the three species is that it is difficult to find an experimental dataset that contains all the necessary information collected under identical conditions. This is important if the objective is to minimize the number of parameters that need to be adjusted between species. Chung et al. [34] collected a series of twitches for trabeculae from the mouse, rat and human, which minimized the variation between conditions and laboratory protocols. That dataset is used here as a reference for the simulations in Figure 2. Unfortunately, Ca^2+^ transients were not recorded at the same time. Thus, to generate Ca^2+^ transients, data from various sources were adapted: (1) for the mouse, data from the study of Ca^2+^ transients observed in mice by Ferrantini et al. [36] were adjusted to the temperature, frequency and magnitude of stimulation in the Chung et al. experiments; (2) for rat trabeculae, we used Ca^2+^ transients observed by Janssen et al. [35] at 25 °C; and (3) for humans, the Ca^2+^ transients were derived from the observations of Ferrantini et al. [37] by adjusting the (peak) magnitude to match the observed tension in Chung et al. [34]. These Ca^2+^ transients used in simulations are shown in the inset of Figure 2.

**Figure 3:**
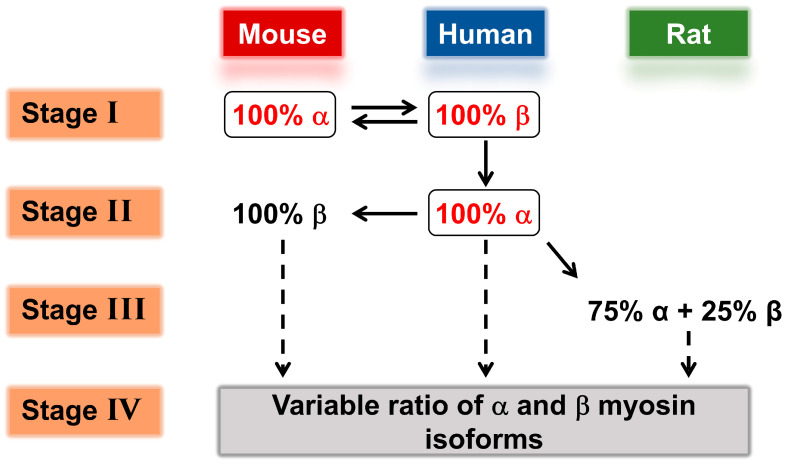
Procedure for modeling twitch contractions of different species. **Stage I**: Based on the previously published mouse twitch, adjusted for the conditions of Chung et al. [34], and the data of Deacon et al. [8] for the crossbridge cycle parameters of mouse α- and human β-myosins. **Stage II**: Based on Stage 1 and the data of Deacon et al. [38] on human α-myosin, which were then extrapolated to mouse β-myosin. **Stage III**: Based on the previous publication on the rat twitch (Mijailovich et al. [1]) and interpolating the crossbridge values for rats between those of mice and humans. **Stage IV**: Using the crossbridge parameter values derived in **Stages I**–**III** to predict the effect of variable ratios of α- and β-myosin in each case.

Figure 3 illustrates our approach to modeling the twitches of each isoform and for each species where we only had a complete set of crossbridge cycle parameters for the human α- and β-myosin and the mouse α. As the first step, the twitches for the mouse (100% α-myosin) and human (assumed initially to be 100% β-isoform for the majority of myocytes) were simulated.

The starting parameters for simulating the tension transients in human and mouse trabeculae were based on the values used in recent simulations of rat and mouse trabeculae [1,15], as listed in Table 1. These core parameters remained constant throughout all the simulations presented here. Note, however, that two parameters differ from those used in the simulation of twitches in mouse trabeculae [15]: the myosin stroke-forward cap-rate constant, $k_{+Pi}^{cap},\;$(Table 1) and the Ca^2+^ sensitivity of $k_{+PS}$, which is the rate constant for the transition out of the parked state (denoted as ${\left[Ca\right]}_{50}$ in Table 1). The value of $k_{+Pi}^{cap}$ was arbitrarily set as a very fast value for the mouse [15]; it does not affect the forward power-stroke rate and is merely set to keep the stochastic process within the numeric bounds. The increased sensitivity of myosin recruitment from the parked state ($k_{+PS}$) for the mouse trabecula from ${\left[Ca\right]}_{50}$ = 1 µM in the rat [1] to 0.6 µM [15] is minor and within the corrections that might be expected for the difference in experimental conditions. Thus, for consistency, in all simulations presented here, we used the same $k_{+PS}\left(Ca^{2+}\right)$ function, i.e., we kept all other relevant constants the same and set ${\left[Ca\right]}_{50}$ to 1 µM (Table 1).

The only parameters adjusted between species and isoforms were the crossbridge cycle state transition rate constants, as listed in Table 2. In the case of human β- and mouse α-isoforms these parameters have, in most cases, been defined in solution with expressed motor domains by Deacon et al. [8], with adjustments for temperature and ionic strength as defined by Mijailovich et al. [50]. Using the parameters listed in Table 2, we were able to achieve a good match between the MUSICO simulations and the observations of Chung et al. [34], including the rate of tension rise, relaxation and the peak tension (see Figure 2).

The values in Table 2 also list the model parameters used to simulate twitches for human 100% β-myosin trabeculae. Compared to the mouse with 100% α-myosin, the changes in the crossbridge cycle parameters for the human simulations, in general, were quite modest. The human simulations required the following changes compared to the values used for the mouse (see Table 2): a five-fold reduction in the rate constant for myosin-binding to actin, $k_{+A}$, a four-fold decrease in the rate of the reverse myosin stroke, $k_{-Pi}^{cap}$, a 15-fold decrease in $k_{+D}$ (limiting rate constant of adenosine diphosphate (ADP) release) and a two-fold reduction in both the forward and reverse rate constants of ATP hydrolysis, $k_{+H}$ and $k_{-H}$. These were similar to the changes measured in the crossbridge cycle kinetics by Deacon et al. [8]. Compared to the values discussed so far, much smaller decreases were needed for the detachment of weakly attached crossbridges, A.M.D.Pi, $k_{-A}$ of ~30%, and the free energy change of the working stroke, $G_{stroke}$ of 13%. Note that when using the Ca^2+^ transient shown in Figure 2, only these seven parameters were adjusted between the mouse and human simulations, with both showing good agreement with experimental observations and providing a good description of the tension transients.

**Figure 4:**
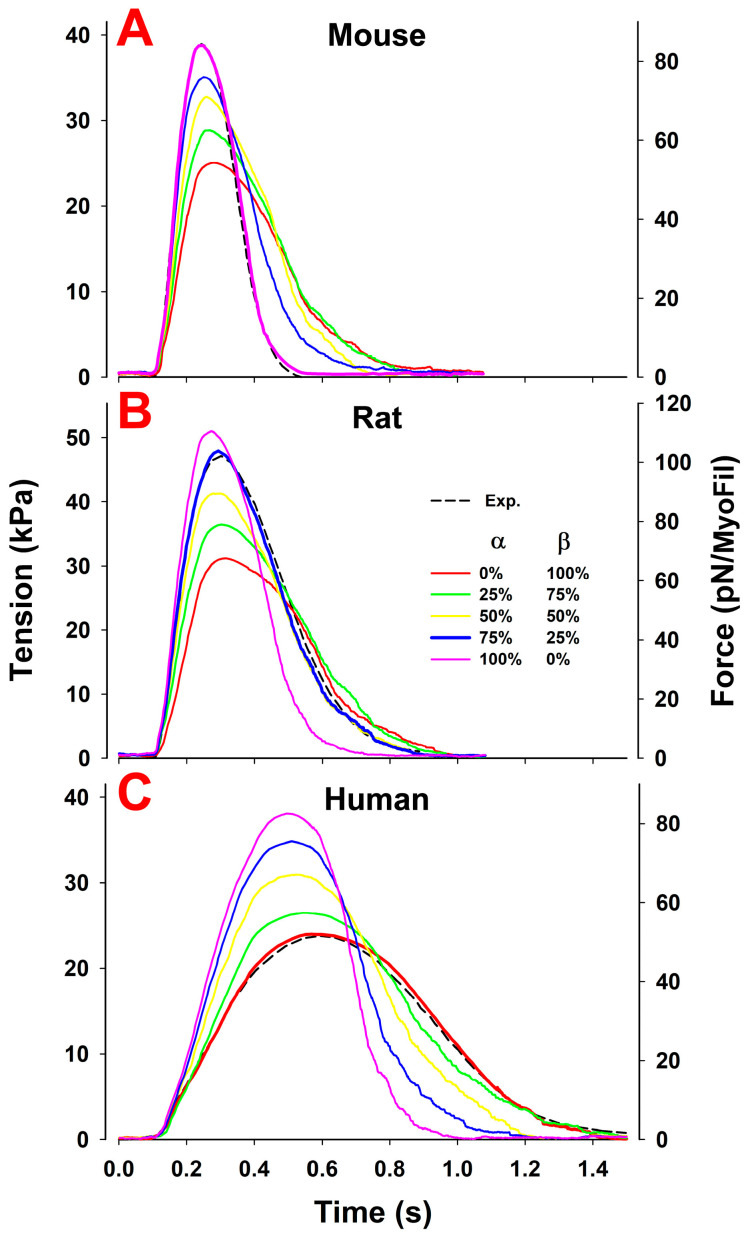
Effect of various fractions of α- and β-isoforms on twitch responses in fixed-end isometric conditions from mouse (**A**), rat (**B**) and human (**C**) trabeculae. The twitches show about the same twitch tension but the mouse (**A**) shows a much faster tension rise and tension relaxation than the rat (**B**). These parameters are much slower in the human for both the α- and β-isoforms (**C**), signifying differences in kinetics parameters of the same isoform across the species (Table 2). These responses were simulated assuming the same Ca^2+^ transient as in Figure 2. The differences between the three species, therefore, also reflect the differences in the magnitude of the peak and duration of respective Ca^2+^ transients.

The simulations could thus describe the mouse twitch with 100% α-myosin and the human twitch with ~100% β-myosin, taking into consideration both the species and isoform differences. To address the differences due only to the myosin isoform, we next performed simulations of the human twitch for the 100% α-myosin isoform. Deacon et al. generated a set of crossbridge parameters for human α-myosin [38], which were used by Johnson et al. [51] to model the complete crossbridge cycle for human α- and β-myosins. This work was consistent, with little change in the rate constants for crossbridge attachment ($k_{+A}$) to and detachment ($k_{-A}$) from the A.M.D.Pi state between α- and β-myosin. In contrast, the ADP release rate constant, which controls the flux of crossbridge detachment following the crossbridge power stroke, and the observed maximum shortening velocity were ~two- to three-fold faster for human α- compared to β-myosin. To maintain a similar duty ratio, the hydrolysis step ($k_{+H}$ and $k_{-H}$), controlling the lifetime of the detached crossbridge, was also observed to be two- to three-fold faster for human α-myosin, which was more similar to that of mouse α-myosin. All parameters that differed between human α- and β-myosin are listed in Table 2. The simulation for 100% α-myosin, with an unchanged Ca^2+^ transient, had a faster tension rise, faster relaxation and higher peak tension than those for the β-isoform (Figure 4C). There are, of course, no experimental data for a human ventricular cardiac cell with 100% α-myosin, but atrial cardiac myocytes have an ~75% α-isoform. Note that atrial cardiac muscle twitches will have a different calcium transient than ventricular twitches, and therefore, cannot be used to predict the twitch for a myocyte with a high α-myosin content until atrial Ca^2+^ transients are available.

There are almost no data characterizing mouse β-myosin. However, when assuming that the differences between isoforms are similar across the three species, we can use the data for mouse α- and human α- and β-myosin to predict the parameters for mouse β-myosin. These estimated parameters for mouse β-myosin are listed in Table 2 and a simulation for a mouse expressing 100% β-myosin is shown in Figure 4A. Similarly, as for human α versus β, the mouse α-isoform has a faster tension rise, faster relaxation and a higher peak tension than the β-isoform.

In contrast to our previous report, which simulated twitch tension transients for rat trabeculae without considering the myosin isoform composition [1], here, a 75/25% ratio of α- to β-myosin was used with a random distribution of the two myosin isoforms along thick filaments and across sarcomeres. Because the parameters for the rat crossbridge cycle are not well-defined, we set starting values for α-myosin that were similar to those used in the earlier rat simulations [1], and for rat β-myosin, we used parameters between those of the mouse and human β-myosins. Specifically, we adjusted the ADP release rate constant ($k_{+D}$) to match the expected differences in shortening velocity of rat vs. mouse and human, and we adjusted the rate of binding to actin ($k_{+A}$) to fall between the values for the mouse and human based on Johnson et al. [51].

The differences between human twitches and the two rodent twitches reflect the changes in species size, differences in Ca^2+^ transients (which reflect physiological heartbeat frequencies), species-specific isoforms and the differences in the ratios of α- and β-myosin isoforms expressed in the ventricle: with 100%, 75% and ~5% α-myosin isoforms for the mouse, rat and human ventricle, respectively. For each species, $k_{+A}$ and $k_{-A}$ do not change between the α- and β-isoforms but do become smaller as the mammals get larger (Table 2). $G_{stroke}$ and $k_{+H}$ vary little between species and are smaller for β- and α-myosin in each case. For $k_{-Pi}$, and $k_{+D}$, the values are two- to three-fold smaller in β- than α-myosin and decrease as the species get larger, i.e., decreasing by ~20% from the mouse to rat and by a further ~80% from the rat to human. The biological significance of these changes will be considered in the Discussion.

### Role of Mixed Myosin α- and β-Isoforms in Cardiac Muscle Twitch Contraction

With a collection of model parameters for the α- and β-isoforms for each of the three species, it is now possible to consider the role of the myosin isoform in mixed α- and β-populations independent of any changes in calcium transients. This is not possible to address experimentally as changes to the isoform content are invariably coupled with adjustments to the Ca^2+^ transients, making the role of each difficult to assess. To explore the effect of the different isoform mixtures, we used the parameter set for human α- and β-isoforms, which are well-defined experimentally (Table 2), to simulate the fractional variations in α- and β-myosin isoforms for human cardiac tissue (Figure 4C). We then repeated the simulations with each of the other species (Figure 4), assuming for the purpose of the simulations that the Ca^2+^ transients specific to each species do not change from those shown in the inset of Figure 2. In all simulations, we used mixed ratios of the two isoform homodimers randomly distributed through the sarcomere.

**Figure 5:**
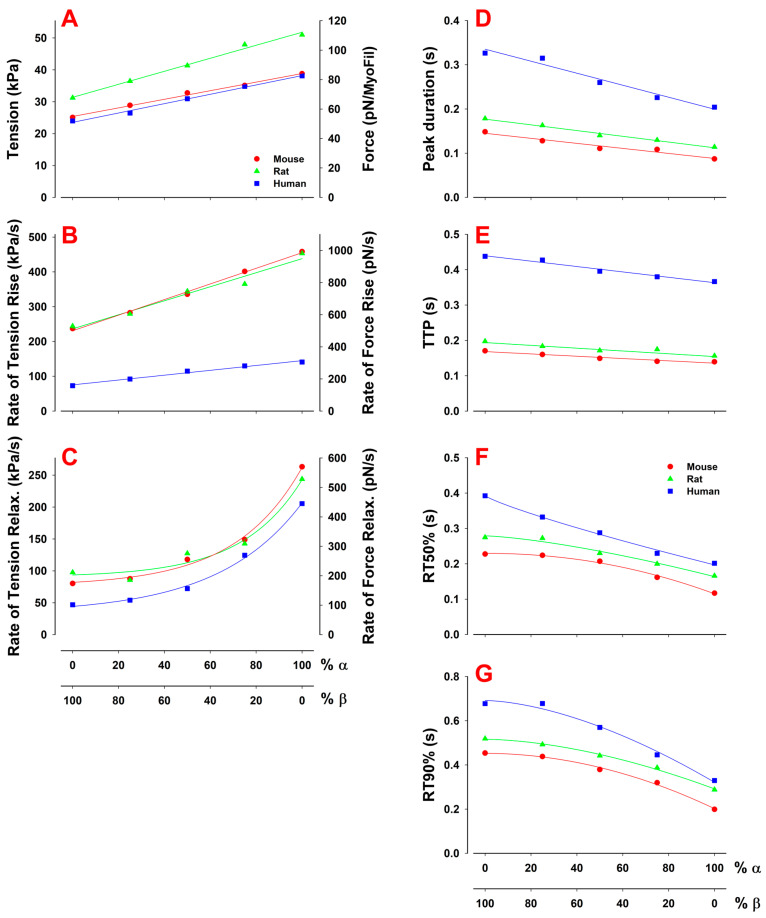
Effects of fractions of α- vs. β-isoforms on twitch characteristics. Parameters plotted are (**A**) peak tension (PT), (**B**) rates of tension rise and (**C**) relaxation, defined as a slope between 30 and 75% of the PT during the rise and vice versa during relaxation, (**D**) peak duration, defined as the time during which the tension is above 90% of the PT, (**E**) TTP, defined as the time from the onset of twitch contraction till the tension reaches the peak, (**F**) RT50% and (**G**) RT90%, defined as the time intervals from the time when the PT is reached to the times at which the tension relaxes to 50% and 90% of the PT, respectively. The magnitude of the peak tension and the rate of tension rise approximately linearly increased with the increase of α-isoform fraction, while the peak duration and TTP linearly decreased as the % α increased. The rate of tension relaxation nonlinearly increased while the RT50% and RT90% nonlinearly decreased.

For the mixtures of α- and β-myosin isoforms in the human (Figure 4C), and similarly, in the mouse and rat isoforms (Figure 4A,B), an increase in the percentage of the α-myosin isoform shows an increase in the peak force and an increase in both the rate of tension rise and relaxation, resulting in a significant reduction in twitch duration (Figure 5D). Recall that the Ca^2+^ transients were assumed to be independent of the ratio of isoform in these simulations. Therefore, the changes in twitch responses are brought about by differences in the myosin isoforms present. Thus, variation of the amount of α- vs. β-myosin has a major effect on both the magnitude and kinetics of the cardiac twitch in each species.

The plots of these, and other key twitch characteristics, for all three species as a function of the myosin isoform composition are shown in Figure 5. Note that some twitch characteristics have an almost linear dependence on the % α-myosin (peak tension (PT), rate of tension rise, time to peak tension and twitch peak duration) while the relaxation characteristics—defined by time intervals from the time when the PT is reached to the time at which the tension relaxes to 50% and 90% of the PT (RT50% and RT90, respectively)—show a distinct nonlinearity. For relaxation, the values were relatively insensitive to the percentage of α-myosin until a 50:50 ratio was reached, and then they changed more dramatically with a further increase in the % α-myosin. For all twitch characteristics reported, the values for the mouse and rat were similar, with the human being more distinct. This was expected as the mouse and rat contractions are similar to each other and reflect closer physiological behavior. The one exception was the peak tension, which was similar for the mouse and human trabeculae and larger for the rat. This probably reflects the higher peak [Ca^2+^] observed (experimentally) in the rat (see Figure 2).

**Figure 6:**
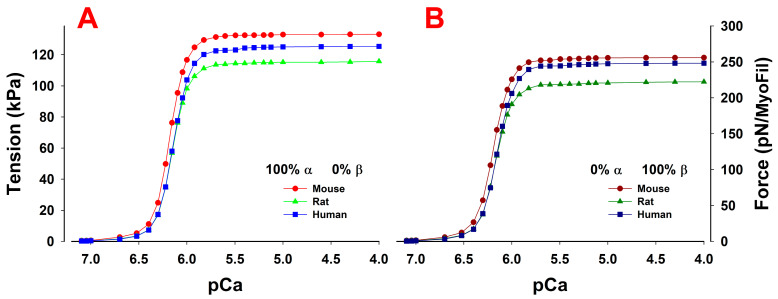
Force-pCa relations of trabeculae containing (**A**) 100% α- or (**B**) 100% β-isoforms in mouse, rat and human cardiac muscles. At full activation at pCa < 5.5, all cases showed similar tension with differences between the species of <20 kPA or <20% of the largest tension, and even less between the corresponding α and β in the same species. For the α- and β-isoforms, the mouse showed the highest and the rat the lowest tension. Interestingly, all showed very similar Hill coefficient n_H_ and pCa_50_ values.

The predicted maximum isometric tension at full activation (pCa < 5.5) was modeled using the same parameters (Table 3). The maximum tension varied only a little between the species (within 10%, Figure 6, Table 3) and was slightly lower for β-myosin in each case. These values fell within the range reported experimentally [41,52,53].

Simulated force-pCa relations also showed that the predicted Ca^2+^ sensitivity was virtually the same across the species and independent of myosin isoforms, as reported experimentally (Table 4) [52]. This was expected because all simulations used the same Ca^2+^-binding rates and affinities, as reported in Table 1. Similarly, the predicted isometric ATPase at full Ca^2+^ activation matched those observed experimentally. Each α-myosin isoform ATPase was two- to three-fold faster than the β-isoform in the same species. The mouse ATPases were 1.5 times the rat values for both α and β, and they were three to four times faster than the human for β-myosin and five to six times faster for α.

Overall, our simulation, based on the human crossbridge parameters, describes human trabecula twitches well, predicts the form of the twitch for 100% α-myosin and presents how the twitch varies with different fractions of α-myosin expressed in the cell. This is important for helping us to understand how myocytes with different % α-myosins contract, how this may influence the contraction of different regions of the human heart—e.g., atrium vs. ventricle and ventricular epi- vs. endocardium—and how the effect of changes to the ratio of α- vs. β-myosin during cardiac development, aging and diseases [12] can influence ventricular systole. Similar exercises for the rat and mouse will help us to translate the results on these model organisms to the human heart, thus increasing the value of rodent models to human (patho)physiology.

## 4. Discussion

The twitch characteristics of cardiomyocytes from model species such as the rat and mouse are quite distinct from those of human cardiomyocytes, making extrapolation from these model systems to humans challenging. Here, we used the explicit 3D modeling platform MUSICO to explore these species differences and defined the minimal number of changes in crossbridge parameters required to simulate twitches from the trabeculae of all three mammalian species (Table 2). We previously simulated the adult rat trabecula twitch [1] and twitches from adult transgenic mouse trabeculae expressing different forms of cTnC [15], but the experimental data were taken from publications from different laboratories where the experiments were performed under slightly different conditions. Here, we use a single data set from one laboratory [34], thus minimizing variations within the dataset.

### Effect of Differences in Myosin Isoforms within and between Species

The differences between the twitch characteristics of the three species are driven by the requirement for very different speeds of cardiac contraction and include many factors such as the action potential, calcium transient (see Figure 2) and differences in the sarcomeric proteins. Here, we used literature data of experimentally measured Ca^2+^ transients in the ventricular muscle of the three species and focused on the specific effects of the myosin isoforms. Differences in myosins occur at two levels, as discussed above. The mammalian heart contains both α- and β-myosin isoforms, but the ratio of the two differs between each species [12]. This ratio can also vary between heart regions, as is the case, for example, between the human ventricle and atrial myocardium and across the ventricular wall from the epicardium to the endocardium. There is also a significant difference in the same isoform across species. This is well-documented for the β-isoform in slow skeletal muscle, which is identical in sequence to the β-isoform in cardiac muscle, where myosin is tuned to the speed of contraction of each species and closely linked to the overall size [13,54]. Similar tuning of the α-myosin isoforms may be expected [54,55]. In contrast to myosin, the thin-filament protein isoforms show little variation and each has high sequence conservation across species. Hence, our focus here is on myosin.

Our results, shown in Figure 2 and Table 2 demonstrate that distinct twitch characteristics can be modeled with alterations to a small set of seven crossbridge parameters linked to the properties of the myosin present. Of these seven parameters, two ($k_{+A}$ and $k_{-A}$) are characteristic of the species—i.e., they do not change between α- and β-isoforms in any of the three species—and three parameters ($k_{-Pi}$, $k_{+H}$ and $k_{-H}$) are characteristic of the isoform—i.e., the value for α is the same or similar across species but varies between the α- and β-isoforms (Table 2). However, note that the ratio of $k_{+H}\;/k_{-H}$ does not vary. The changes in $G_{stroke}$ (−11.3 to −13 k_B_T) are small across all species and isoforms. This leaves just $k_{+D}$, which changes substantially for all species (five- to seven-fold) and myosin isoforms (two- to three-fold). It is important, therefore, to understand how each parameter contributes to the overall contraction characteristics in each case. This will help explain how contraction can be altered by varying the ratio of α- to β-isoforms (see Figure 4 and Figure 5) or altered by evolutionary changes to isoform sequences to match the requirements of each species, such as different body/heart sizes and heart rates. The two primary characteristics of α- vs. β-myosin isoforms are the faster ATPase and contraction and lower economy of energy usage by α-myosin [12].

In Figure 4 and Figure 5 we demonstrate how varying the ratio of α- to β-isoforms changes the twitch characteristics in each species, assuming a constant Ca^2+^ transient. In each case, as the proportion of the faster α-isoform increases, there are increases in the rates of tension rise and relaxation, resulting in an increase in peak tension and reduction in tension duration (tension time integral) [56,57]. Note that these changes are independent of the Ca^2+^ transient. The changes are, therefore, linked to changes in $k_{-Pi}$ and $k_{+H}$ + $k_{-H}$ (linked to occupancy of the force holding state) and $k_{+D}$ (linked to the exit from the force holding state), which differ two- to three-fold between the two isoforms. In a dynamic cycle such as that here, it is not possible to say that $k_{-Pi}$ and $k_{+H}$ + $k_{-H}$ control the tension rise while $k_{+D}$ controls relaxation as an alteration in either set of constants affects the balance in the cycle. This is demonstrated in Supplementary Materials Figures S1 and S2, where a set of simulations is presented in which $k_{+D}$ is varied and affects both the rate of tension rise and relaxation through the resulting changes in the duty ratio, and hence, isometric force (see Mijailovich et al. [50] and Johnson et al. [58]). For this reason, it is likely that changes in the exit from ($k_{+D}$) and entry into ($k_{-Pi}$ and $k_{+H}$ + $k_{-H}$) the force holding states remain in balance, allowing the duty ratio and isometric force to remain the same while altering the overall cycling rate of crossbridges.

This ability to alter contraction parameters is important not only for different species to tune their contraction parameters by varying the basal ratio of expressed isoforms but also, as discussed above, for varying contraction in the atria vs. ventricle, across the ventricle wall and in response to physiological changes such as hydrodynamic stress. Variation in the ratio of α- to β-isoforms also occurs in end-stage heart failure where α-isoform expression is reduced [4] and some recovery of function is observed if the α-isoform expression can be upregulated [59].

Moss and colleagues discussed the potential advantages of the expression of variable levels of α across the heart wall and suggested that a faster contraction of the outer heart wall (with higher α-isoform expression) could result in a more efficient ejection of blood from the ventricle [60,61]. They predicted that expression of a low level (10%) of α could increase the rate of tension rise while the predominance of β would maintain a slower rate of relaxation. This is consistent with our modeling shown in Figure 5 where increasing α to 20% has little effect on relaxation rates, while the rate of tension rise and the peak tension increase for each of the species.

To examine how mouse trabeculae contraction with 100% α-isoform differed from that of human with 100% α, we used the derived model parameters (Table 2) and applied the Ca^2+^ transient of the mouse to the human and vice versa (Supplementary Materials Figure S3). Comparing the 100% mouse α-myosin tension transient with that of the 100% human α (Supplementary Materials Figure S3A,C) defined how the parameters of the α-isoform control the twitch characteristics. The human twitch is much slower (rise time, time to peak and relaxation) than the mouse with a similar peak force. Since the Ca^2+^ transient is the same, these changes are due to alterations of $k_{+A}$, $k_{-A}$ (controlling binding in the weakly attached pre-force state) and $k_{+D}$ (exiting from the force holding state). The equilibrium constant $K_{A}\left(=k_{+A}\;/k_{-A}\;\right)$ varies between species ($K_{A}$ = 6 for the mouse and 1.5 for the human; Table 2), and thus, the occupancy of the weakly attached state will be greater for the mouse, in addition to forming faster. Increased occupancy of the weakly attached state will then lead to an increase in the rate of entry into the subsequent force holding states. As mentioned above, it is too simplistic to say that $k_{+A}$ and $k_{-A}$ control the rate of tension rise while $k_{+D}$ controls the relaxation as each step contributes to the overall balance of the cycle (Supplementary Materials Figures S1 and S2, and [50,58]). It is of interest that the different isoforms change the cycle speed by altering $k_{+D}$, $k_{-Pi}$ and $k_{+H}$ + $k_{-H},$ while between species, it is $k_{+D}$, $k_{+A}$ and $k_{-A}$. Both approaches maintain the duty ratio, and hence the isometric force, while changing the cycling speed that alters the economy of force generation.

Mouse and human ventricles predominantly express a single myosin isoform. In contrast, rat ventricles express 75% α and 25% β. The spatially explicit MUSICO platform allows the modeling of the presence of mixed isoforms in the sarcomere. In all simulations presented here, we assumed a random mixture of myosin isoform homodimers in each thick filament and across each sarcomere. We used estimates of the properties of rat α- and β-isoforms (Table 2) to generate model predictions that describe the twitch quite well (Figure 2). The set of parameters used in the rat twitch is in good agreement with those used in our earlier paper, which treated the myosins as a single population, denoted as Myo_eq_ in Supplementary Materials Table S1, which compares the parameters used in each study. Note that the data collected from twitches in rat trabeculae [35] were obtained in slightly different conditions than in the experiments used in this study [34] and that the parameters extracted from simulations reported in [15] were obtained by fitting the experiments performed at 22.5 and 27.5 °C but not at 25 °C.

## 5. Materials and Methods

### 5.1. MUSICO Model Parameters

The model parameters used in simulations can be categorized into two groups: (1) parameters that are the same across the species, summarized in Table 1, and (2) parameters specific to the mouse, rat and human muscle, as well as specific isoforms present in the muscle samples, which are shown in Table 2. Specifically, the parameters used in all simulations are: the geometry of the sarcomere, the elasticity of the thick and thin filaments, the kinetic parameters defining thin filament regulation by [Ca^2+^] and several parameters of the crossbridge cycle.

*Geometry of the sarcomere*: Each myosin filament contains 100 crowns, i.e., 300 myosin molecules with a length of ~1.58 μm [62]. Half of the crowns are located on each side of the sarcomere. The crowns, axially spaced at 14.3 nm apart, form a region of the thick filament populated with crossbridges that can interact with neighboring thin filaments. Actin filaments have variable lengths, and the distribution of lengths depends on the cardiac muscle type and likely on species, but for simplicity, we assume the range of 0.6 to 1.1 μm (obtained from a rat atrial trabecula) [27]. The monomer spacing in the relaxed thin filament is ~2.736 nm and the half period of each strand is 35.56 nm [48,63,64]. In all simulations, the sarcomere lengths (SL) are set at 2.2 μm following the experiments of Chung et al. [34]. The value of the interfilament spacing $d_{1,0}$ for a trabecula at SL 2.2 μm is set at ~33.83 nm. The interfilament spacing is an important factor in the modulation of myosin-binding to actin, and possibly, other rates in the crossbridge cycle [22,65].

*Elasticity of the thick and thin filaments, and titin*: The extensibility of the filaments is defined by the filament moduli (elastic modulus × cross-section area), which is derived from X-ray diffraction or direct measurement: for the thin filament, $K_{a}=A_{a}E_{a},$ and for the thick filament, $K_{m}=A_{m}E_{m},$ as taken from [48,49]. The modulus, AE, is used here instead of the stiffness, AE/L, because the reported stiffness values depend on the filament length, L, and cross-section area, A, which are not well-defined for the filaments. The nonlinear elasticity of titin is adopted from [22] and used in all simulations. Moreover, following the approach of Mijailovich et al. [1], the series elasticity of trabeculae is derived from the experiments of Caremani et al. [28] and used in all simulations.

*Ca^2+^ regulation of thin filaments*: The kinetics of Ca^2+^-binding to cTnC, the affinity of cTnI to cTnC or cTnI to actin and the elasticity of CFC are assumed to be the same across different species. The forward constant of Ca^2+^-binding to cTnC is defined as $k_{Ca}\left(Ca^{2+}\right)={\tilde{k}}_{Ca}\cdot \left[Ca^{2+}\right]$ and the dissociation constant as $k_{-Ca}$. The affinities of cTnI-cTnC and cTnI-actin are defined by the equilibrium rate, λ, and cooperativity coefficient, $\varepsilon_{o}$. For the CFC model parameters ($\phi_{-}$, $\phi_{o}$, $\phi_{+}$, $\sigma_{0}$ and 1/ξ), we used those reported in [1].

*Biochemical parameters for the crossbridge cycle*: The parameters needed to define the eight-state biochemical ATPase cycle for the mouse-α, the human-α and -β and the bovine β-isoforms were measured by Deacon et al. [8] and then used by Mijailovich et al. [50] and Johnson et al. [58] to model the crossbridge cycle and predict the unloaded motility, isometric force and load dependence of the cycle. We, therefore, have a precise definition of the cycle for the α- and β-isoforms from two different mammals. Generating starting values for the transition rates between the five states in the crossbridge model used here was based on these values.

Translation of values between the rat, mouse and human was based on the observation that the ATPase cycle, motility and muscle-shortening velocity vary with the mammal size [13], as is also reflected in the twitch parameters, i.e., the rates of activation and relaxation measured in the trabeculae, myocytes or myofibrils. For example, the mouse has ~1.5–2-fold faster rates than the rat, and the rat has two to four times faster rates than the human. Similarly, measurements on the α- and β-isoforms from the same species show that the same parameters create a two- to four-fold increase for the α- over β-isoform values. In contrast, the isometric force measured in single-molecule assays or in myofibrils or cellular systems shows little change. Based on these assays and the values we previously used for rat and mouse twitch simulations, we generated the parameters for our six-state crossbridge model (Table 2).

In addition, the details of the fitting process required information concerning the series of elastic elements in the muscle, which allow internal sarcomere shortening during contraction and elongation during relaxation, and the presence of a parked state analogous to the SRX state. These were described in our previous work (Mijailovich et al. [1,15]) and outlined in the Methods section. Here, we extended this approach to human cardiac muscle, which predominately expresses the β-myosin isoform.

### 5.2. MUSICO Software Environment and Simulation Details

The MUSICO platform was developed as a C++ object-oriented application that includes the LAPACK linear algebra package and the deal.II finite element library. The typical run times for these simulations depend on the numbers of actin and myosin filaments. For example, a simulation of 200 myosin filaments over 1 s with a time-step of 10 μs requires ∼3.5 h on the Intel Xeon E5-2670@2.6GHz 8-core processor with 16 GB of RAM.

To estimate the model’s free parameters in the stochastic simulations, we used a half-sarcomere with 200 (half) myosin and 400 actin filaments, which was sufficient for statistical averaging without requiring us to run the simulation multiple times. In the final simulations, we increased the number of (half) myosin filaments per half-sarcomere to 500 as an approximate number of the filaments in a cross-section of a typical myofibril.

## 6. Conclusions

Computational simulations can be of great value in addressing questions that are intractable or difficult to assess quantitatively by experimentation. One such question is how we can understand the differences between the cardiac contractions in small rodents and humans. These can be attributed to both the expression of different isoforms and the adaptation of each protein isoform to the requirements of different body sizes. Both issues are found in myosin isoforms of the heart (α and β), which are distinct in their mechanical and biochemical properties between α- and β-isoforms and between the same isoform properties in each species. Each mammal also varies in terms of the α:β isoform ratio expressed in the heart. Therefore, understanding the role of each isoform is difficult to address experimentally.

Here, we used the spatially explicit MUSICO platform to simulate twitches in trabeculae from the mouse, rat and human. This allowed us to identify the minimal number of parameter changes (only seven) needed to simulate each type of twitch and attribute the changes to both α- and β-isoforms differences and to differences between the three mammals.

Of the seven parameters, three ($k_{-Pi}$, $k_{+H}$ and $k_{-H}$) are features of the specific isoform (i.e., α- or β-isoform), two ($k_{+A}$ and $k_{-A}$) are specific to the species, one ($k_{+D}$) differs both between the isoform and between species and the last parameter ($G_{stroke}$) requires only moderate changes to match the experimental data.

When both isoforms are expressed in myocytes, the twitch rise time is proportional to the % of α-isoform present, while the relaxation time reflects the properties of the β-isoform unless α is >50% of the expressed isoform. The twitch peak force depends on the isoform fractions (Figure 4), but the maximum force at full activation is relatively independent of the isoform mix (Figure 6).

The exact form of twitch contraction is a combined effect of both the myosin isoform properties and the Ca^2+^ transient. This allows the twitch to be modulated in the short term by altering the Ca^2+^ transient to adapt to altered demands. However, altering the Ca^2+^ transient over a long period could alter the ATP requirements of the cardiomyocyte and thus affect the overall energy balance of the cell. On a longer timescale, the twitch can be adjusted by altering the myosin isoform expression while maintaining the energy balance.

Understanding how varying the level of isoform expression affects the twitch is important to understand why there are different levels of myosin expression across the heart and how alterations to myosin expression occur in heart failure and other pathologies—e.g., how much compensation can arise through a myosin isoform shift vs. changes in calcium transients or post-translational modifications of sarcomere proteins (phosphorylation, acetylation, etc.). The work presented here demonstrates how computational modeling helps to define the different contributions of each element. This approach could have strong applications in enhancing drug developments and significantly reducing the costs of newly developed therapeutics.

## Figures and Tables

**Table 1:** Common MUSICO parameters for all simulations of twitch contractions in intact trabeculae at fixed lengths and a temperature of 25 °C.

Description	Parameter	Iso Trabeculae
**Crossbridge Cycle**		
Working stroke [1,21,22,39]	d	10.5 nm
Second working stroke [1,21,22]	δ	1 nm
Myosin stroke forward cap rate constant	$k_{+Pi}^{cap}$	1000 s^−1^
ATP-binding and myosin detachment rate constant ^a^	$k_{+T}$	10^6^ s^−1^
Crossbridge stiffness [1,21,22,39]	κ	1.3 pN/nm
$k_{B}T$ at 25 °C	$k_{B}T$	4.116 pN·nm
**Parked State**		
Transition rate constant to “parked state” ^b^	$k_{-PS}$	200 s^−1^
Baseline rate constant ^b^	$k_{PS}^{0}$	5 s^−1^
Amplitude ^b^	$k_{PS}^{max}$	400 s^−1^
Calcium Hill function slope ^b^	b	5
Half activation point of the Hill function ^b^	${\left[Ca\right]}_{50}$	1 μM
**Calcium Kinetics**		
Calcium-binding to cTnC equilib. rate constant [30,40]	${\tilde{K}}_{Ca}$	10^6^ M^−1^
Calcium-binding rate constant to cTnC [30,40]	${\tilde{k}}_{Ca}$	7.54·10^7^ M^−1^·s^−1^
Calcium dissociation rate constant from cTnC [41,42,43]	$k_{-Ca}$	75.4 s^−1^
cTnI-actin equilibrium rate const. at high Ca^2+^	λ	10
cTnI-actin backward rate const.	$\lambda_{-}$	375 s^−1^
cTnI-actin-Ca cooperativity coefficient [30,40]	$\varepsilon_{o}$	0.01
**CFC**		
Tropomyosin pinning angle [44]	$\phi_{-}$	−25°
Myosin Tm angular displacement [44]	$\phi_{+}$	10°
Angular standard deviation of free CFC [25,45]	$\sigma_{0}$	29.7°
Persistence length of Tm-cTn confined chain [25]	1/ξ	50 nm
**Sarcomere**		
Length of sarcomere	SL	2.2 μm
Reference length of actin filament [27,46]	$L_{a}^{o}$	1.1 μm
Interfilament spacing at SL = 2.2 μm [47]	$d_{10}$	33.8 nm
Thin filament elastic modulus [48,49]	$AE_{a}$	65 nN
Thick filament elastic modulus [48]	$AE_{m}$	132 nN

^a^ Based on mouse and human α-myosin values in [8,38], with corrections for temperature and ionic strength as documented in [50]. ^b^ Assumed.

**Table 2:** Variable crossbridge cycle model parameters for different species and myosin isoforms.

Description	Parameter	Mouse		Rat		Human	
		α	β	α	β	α	β
Myosin-actin binding rate	$k_{+A}^{o}$ (s^−1^)	330	330	160	160	60	60
Myosin-actin detachment rate ^a^	$k_{-A}^{o}$ (s^−1^)	55	55	40	40	40	40
Power-stroke energy change ^c^	$G_{stroke}$ (k_B_T)	−13	−11.3	−13	−11.3	−12.5	−11.3
Myosin reverse-stroke cap rate ^b^	$k_{-Pi}^{cap}$ (s^−1^)	40	13	33	11	30	10
ADP release rate ^a^	$k_{+D}^{o}$ (s^−1^)	150	50	120	40	22	10
Hydrolysis forward rate ^a^	$k_{+H}$ (s^−1^)	150	70	150	63	150	30
Hydrolysis backward rate ^a^	$k_{-H}$ (s^−1^)	15	7	15	6.3	15	5

^a^ Based on mouse and human α- and β-myosin values in [8,38,50], with corrections for ionic strength as documented in [50] and temperature as documented in [1]. ^b^ The power stroke rates ($k_{+Pi}$ and $k_{-Pi}$) were expected to be slower in β-isoforms by ~five-fold, and this was achieved by reducing the adapting $G_{stroke}$ and decreasing the $k_{-Pi}^{cap}$ by a factor of ~3. For the same isoform, the power-stroke rate increase from humans to rats and to mice was accomplished by increasing the $k_{-Pi}^{cap}$. ^c^ Relevant references for $G_{stroke}$ [1,21,22].

**Table 3:** Isometric tension (kPa) at [Ca^2+^] = 10 µM.

	α-Myosin	β-Myosin
**Mouse**	133.1	118.1
**Rat**	115.8	102.6
**Human**	125.4	114.5

**Table 4:** Force-pCa relations for α- and β-myosin isoforms across the species.

	Hill Coefficient		pCa_50_		ATPase (s^−1^)	
	100% α	100% β	100% α	100% β	100% α	100% β
**Mouse**	4.91	4.70	6.18	6.19	2.25	0.90
**Rat**	5.00	4.87	6.15	6.17	1.56	0.64
**Human**	4.86	4.78	6.14	6.15	0.43	0.23

ATPase for rat (75% α–25% β) is 1.32 (s^−^^1^).

## Data Availability

All the data are presented in the manuscript.

## Supplementary Materials

The following are available online at: https://www.mdpi.com/article/10.3390/ijms23031135/s1.
