# Supplementary material for: Effect of Myosin Isoforms on Cardiac Muscle Twitch of Mice, Rats and Humans
Source: Int J Mol Sci. 2022 Jan 20;23(3):1135. doi: 10.3390/ijms23031135 (PMC8835009; doi:10.3390/ijms23031135)
Supplement: Supplementary file 1 [file ijms-23-01135-s001.zip › ijms-1493334-supplementary.pdf]

Supplementary Material

1. *Effect of ADP release rate on twitch characteristics*

Twitch responses to the calcium transients in the trabeculae of different species depend on several crossbridge cycling rates. The most significant parameter controlling the velocity of shortening and the duty ratio is the rate of ADP release (k_{+D}). To examine how k_{+D} contributes to the characteristic features of the twitch responses, we performed simulations for values of k_{+D} in the range from 10 to 300% of the value of k^{o}_{+D} for β-myosins of each of the species listed in Table 2. The peak twitch tension, fully developed isometric tension, duty ratio and characteristics defining the shape of the tension transients are plotted in Figure S1 as a function of $^{*}k_{+D}$, where $^{*}k_{+D}$ denotes a fraction k^{o}_{+D} in (%) and k^{o}_{+D} is specified for each of the species (Table 2). In Figure S2, the transients are plotted as functions of the absolute value of k_{+D} to quantify the change of the twitch parameters solely in relation to the value of k_{+D}. In all cases, the twitch characteristics were sensitive to values below 100% k^{o}_{+D}, but changed little for k^{o}_{+D}> 100%. Moreover, some parameters (e.g., rate of tension rise and rate of relaxation) of the human twitch were less sensitive to k_{+D} than in the two rodent species The absolute k_{+D} is relevant as it shows how the twitch responses are affected by variations in the rates of crossbridge cycling associated with the specific isoform in each of the species.

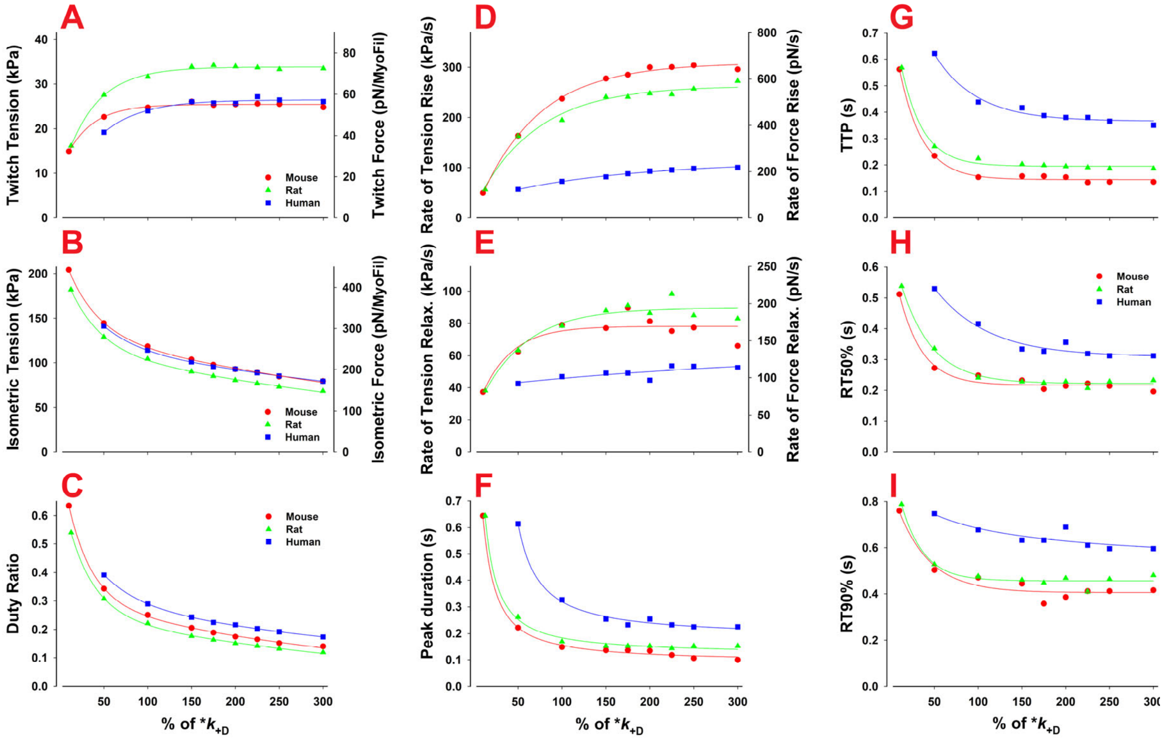

Figure S1. Sensitivity analysis of the effect of change of k_{+D} on twitch peak tension (PT) and other twitch characteristics in mouse, rat and human trabeculae. Rate of ADP release, $^{*}k_{+D}$ and all other parameters are taken to be the same as in the simulations for β-myosins shown in Figure 1. The effects of changes to the %$^{*}k_{+D}$ are shown on the twitch peak tension, isometric tension (PT) at full activation, duty ratio, rate of tension rise (from 75 to 30% of PT) and tension relaxation (from 75 to 30% of PT) during twitch contraction, as well as the peak duration, i.e., the time when tension was above 90% of the PT, the time from the onset of tension rise to the PT time (TTP) and the times to a 50% and 90% fall from PT (denoted as RT50% and RT90%, respectively).

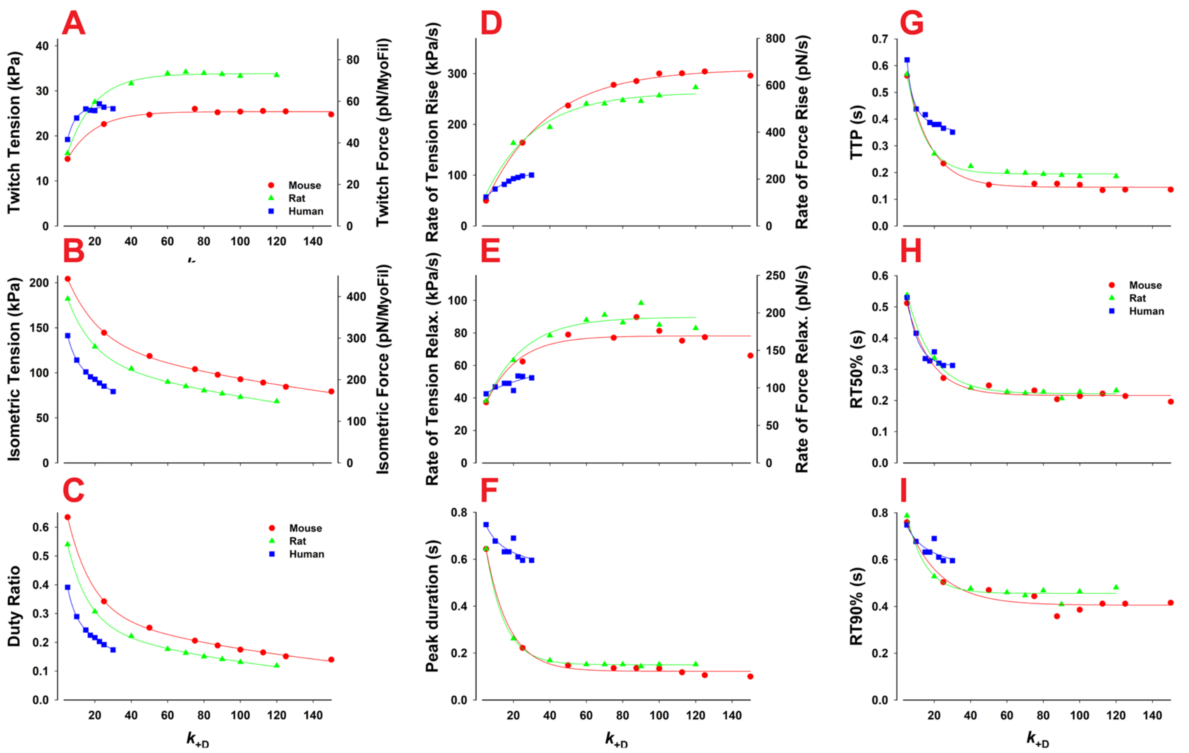

Figure S2. The same data analysis as shown in Figure S1 but here the twitch responses are plotted, for comparison, against the absolute values of ADP release rate, k_{+D} (s^{-1}), for all species. These data show changes in twitch characteristics with an increase of k_{+D} in a variety of ways. In some cases, the characteristics show small differences between the species for the same values of k_{+D}, all falling within a certain range of values (A, G, H and I). Nevertheless, in two cases, the characteristics show a similar pattern but different values with increases of k_{+D} each time (B and C). Interestingly, the peak duration (F) coincides for the mouse and rat but drastically differs for humans.

2. *Effect of Ca^{2+} transient on twitch responses for myosin isoforms observed in different species*

The role of the Ca^{2+} transient in different myosin backgrounds is illustrated in Figure S3. First, using the slower Ca^{2+} transient from the human trabecula, we simulated the response in rat and mouse muscles containing a 100% α- or 100% β-myosin isoform. The changes in twitch tension transients are shown in Figure S3A,B. Similarly, using the mouse Ca^{2+} transient, we simulated twitch tension transients containing 100% human α (Figure S3C). In all cases, the α-isoform shows higher peak tension and a narrower peak than the β-isoform for the same Ca^{2+} transients (Figure S3). The predominately α isoform tension transient in the mouse and predominately β-isoform tension transient in the human (pink line in A and red line in C) closely followed the experimental traces (black dashed lines), while in the rat, the predicted α- and β-isoform transients overestimated (pink line in B) or underestimated (red line in B) the observed tensions (black dashed line), signifying the relevance of the observed mixed composition of α- and β-myosin isoforms in rat cardiac muscles. The slower Ca^{2+} transient from the human trabecula, even with the mouse and rat β-isoforms, causes a larger peak tension and prolonged tension transient but does not affect the rate of tension rise in both rodent trabeculae. Conversely, simulating the tension transient for 100% human α myosin using the much faster mouse Ca^{2+} transient generated a faster but smaller tension transient with a significantly lower peak and much shorter duration.

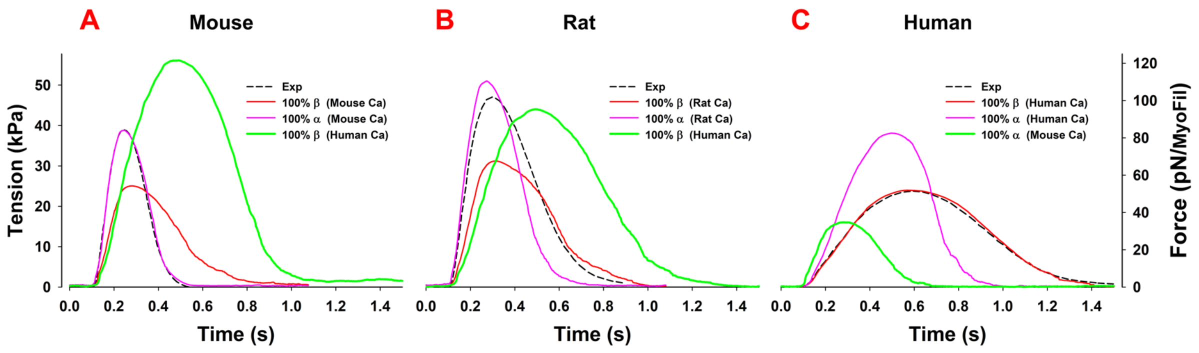

Figure S3. Simulations of twitch tension responses to Ca^{2+} transients from mouse, rat and human trabeculae. (A) The responses of mouse trabeculae containing only an α- or β-myosin isoform to the mouse Ca^{2+} transient; for comparison, the response is also shown of a mouse trabecula containing only β-myosin to a Ca^{2+} transient from a human. (B) The same as in A but in the rat trabeculae containing 100% α- or 100% β-myosin isoforms, the Ca^{2+} transients are from a rat or human. (C) Tension responses of human trabeculae containing only 100% α- or 100% β-myosin to human Ca^{2+} transients and of a human 100% α-myosin trabecula to a mouse Ca^{2+} transient. All Ca^{2+} transients are taken to be the same as those in the simulations shown in Figure 1.

3. *Comparison of parameters used in rat trabeculae twitch simulations using mixtures of α- and β-myosin isoforms and the equivalent myosin isoform used in Mijailovich et al.* [1]

The set of parameters used in the rat twitch simulations is in good agreement with those used in our earlier paper, which treated the myosins as a single population with equivalent kinetic characteristics (Myo_{eq}), as reported in Mijailovich et al. [1]. In Table S1, we list the parameters used in each study. The two sets of experimental data were collected under slightly different conditions; for example, the data from Chung et al. [2] were collected at 25 °C while Jansen et al. [3] reported data at 22.5, 25.0, 27.5 and 30.0 °C. However, Mijailovich et al. performed simulations only at three temperatures, 22.5, 27.5 and 30.0 °C [1], and not at 25.0 °C. Comparing the equivalent parameters used in simulations at 22.5 and 27.5 °C [1] to the rates for α- and β-myosin isoforms, to obtain from the best fits of Chung et al. the tension transients at 25 °C, showed that all equivalent rates (Myo_{eq}), rescaled to 25 °C, were between the rates of α- and β-myosin (see Table S1). It is expected that the equivalent rates are closer to the rates of the α- than the β-myosin isoform. This apparent deviation, as for example in k^{o}_{+D}, can be explained by differences between the observed tension transients of Chung et al. [2] and Janssen et al. at 25 °C [3]. These transients primarily differ in their rate of relaxation, and these rate differences could be attributed to the differences in experimental conditions, and in addition, use of the Ca^{2+} transient from [3] could lead to an approximation.

Table S1. Differences in crossbridge cycle parameters using 75/25 % mixture of α- and β-myosin isoforms as observed in rat trabeculae and the rates for an equivalent single isoform (used in simulations of Janssen's experiments at 22.5 and 27.5 °C [3]).

[a] In simulations of twitch contractions in rat trabeculae at 27.5 °C and 22.5 °C, as reported in Mijailovich et al. [1], a single set of rate constants was used, denoted as the equivalent myosin isoform rates ($Myo_{eq.}$), which combined the effects of the α- and β-myosin isoforms.

**Description**	**Parameter**	25 °C		27.5 °C[a]	22.5 °C[a]
		$\boldsymbol{\alpha}$	**β**	**Myo$_{eq.}$**	**Myo$_{eq.}$**
Myosin-actin binding rate	k^{o}_{+A} (s^{-1})	160	160	226	140
Myosin-actin detachment rate	k^{o}_{-A} (s^{-1})	40	40	46	22
Power-stroke energy change	G_{stroke} (k_BT)	-13	11.3	-13	13
Myosin reverse-stroke cap rate	k^{cap}_{-Pi} (s^{-1})	33	11	10	10
ADP release rate	k^{o}_{+D} (s^{-1})	120	40	60	40
Hydrolysis forward rate	k_{+H} (s^{-1})	150	63	100	100
Hydrolysis backward rate	k_{-H} (s^{-1})	15	6.3	10	10

References

1. Mijailovich, S. M.; Prodanovic, M.; Poggesi, C.; Geeves, M. A.; Regnier, M., Multiscale Modeling of Twitch Contractions in Cardiac Trabeculae. *Journal of General Physiology* **2021,** 153 (3), e202012604.
2. Chung, C. S.; Hoopes, C. W.; Campbell, K. S., Myocardial relaxation is accelerated by fast stretch, not reduced afterload. *J Mol Cell Cardiol* **2017,** 103, 65-73.
3. Janssen, P. M.; Stull, L. B.; Marban, E., Myofilament properties comprise the rate-limiting step for cardiac relaxation at body temperature in the rat. *Am J Physiol Heart Circ Physiol* **2002,** 282, (2), H499-507.
